# Vitamin D deficiency and supplementation in pregnancy in a multiethnic population-based cohort

**DOI:** 10.1186/s12884-016-0796-0

**Published:** 2016-01-19

**Authors:** Åse R. Eggemoen, Ragnhild S. Falk, Kirsten V. Knutsen, Per Lagerløv, Line Sletner, Kåre I. Birkeland, Anne K. Jenum

**Affiliations:** Department of General Practice, Institute of Health and Society, University of Oslo, Postboks 1130 Blindern, N-0318 Oslo, Norway; Oslo Center for Biostatistics and Epidemiology, Oslo University Hospital, Oslo, Norway; Department of Child and Adolescence Medicine, Akershus University Hospital, Lørenskog, Norway; Department of Endocrinology, Morbid Obesity and Preventive Medicine, Oslo University Hospital, Oslo, Norway; Institute of Clinical Medicine, Faculty of Medicine, University of Oslo, Oslo, Norway

**Keywords:** Vitamin D, Deficiency, Supplementation, Pregnancy, Ethnic minority

## Abstract

**Background:**

To investigate ethnic differences in vitamin D levels during pregnancy, assess risk factors for vitamin D deficiency and explore the effect of vitamin D supplementation in women with deficiency in early pregnancy.

**Methods:**

This is a population-based, multiethnic cohort study of pregnant women attending Child Health Clinics for antenatal care in Oslo, Norway. Serum-25-hydroxyvitamin D [25(OH)D] was measured in 748 pregnant women (59 % ethnic minorities) at gestational weeks (GW) 15 (SD:3.6) and 28 (1.4). Women with 25(OH)D <37 nmol/L at GW 15 were for ethical reasons recommended vitamin D_3_ supplementation. Main outcome measure was 25(OH)D, and linear regression models were performed.

**Results:**

Severe deficiency (25(OH)D <25 nmol/L) was found at GW 15 in 45 % of women from South Asia, 40 % from the Middle East and 26 % from Sub-Saharan Africa, compared to 2.5 % in women from East Asia and 1.3 % of women from Western Europe. Women from South Asia, the Middle East and Sub-Saharan Africa had mean values that were −28 (95 % CI:-33, −23), −24 (−29, −18) and −20 (−27, −13) nmol/L lower than in Western women, respectively. Ethnicity, education, season and intake of vitamin D were independently associated with 25(OH)D. At GW 28, the mean 25(OH)D had increased from 23 (SD:7.8) to 47 (27) nmol/L (*p* < 0.01) in women who were recommended vitamin D supplementation, with small or no change in women with sufficient vitamin D levels at baseline.

**Conclusions:**

Vitamin D deficiency was prevalent among South Asian, Middle Eastern and African women. The serum levels of 25(OH)D increased significantly from GW 15 to 28 in vitamin D deficient women who received a recommendation for supplementation. This recommendation of vitamin D supplementation increased vitamin D levels in deficient women.

**Electronic supplementary material:**

The online version of this article (doi:10.1186/s12884-016-0796-0) contains supplementary material, which is available to authorized users.

## Background

Vitamin D deficiency in pregnancy is prevalent [1], especially in women with limited access to sunlight due to minimal outdoor activity or heavy use of sunscreen, cultural practices or traditional clothing, and among women with dark skin pigmentation. In Europe, vitamin D deficiency is reported to be prevalent among pregnant ethnic minority women in the Netherlands [2] and UK [3], but is also observed in the majority population [3–5]. Little is known about the prevalence of vitamin D deficiency among Asian and African pregnant women living in Northern Europe, although a few small studies from Sweden [6, 7] and Norway [8] report a high prevalence of vitamin D deficiency among Somali and Pakistani women. There are few population-based studies exploring vitamin D deficiency in pregnancy in today’s multiethnic Europe, and little is known about the impact of socioeconomic status or the effect of vitamin D supplements [9, 10]. Also studies of multiethnic populations in Australia have uncovered maternal country of birth in Asia and Africa as risk factor of vitamin D deficiency in pregnancy [11].

Studies provide inconsistent and conflicting evidence for adverse maternal and child health outcomes related to vitamin D deficiency [11–16]. However, the current state of evidence suggests unclear benefits of routine vitamin D supplementation for most maternal or child health outcomes [17]. Most studies report results from interventions in non-deficient women. Despite the controversies related to adverse outcomes, and lack of evidence of clear benefits of routine supplementation, most Western countries recommend vitamin D supplementation during pregnancy.

The aims of this paper were to investigate ethnic differences in vitamin D levels during pregnancy, assess risk factors for vitamin D deficiency and explore the effect of vitamin D supplementation in women identified with low values during early pregnancy.

## Methods

### Design, setting and study population

Data are from the STORK Groruddalen project, which is a population-based, prospective cohort of 823 healthy women from 65 countries attending Child Health Clinics (CHC) for antenatal care in Groruddalen, Oslo, Norway, between May 2008 and March 2010. Groruddalen covers affluent as well as relatively economically deprived residential areas, and has a population with diverse socio-economic status. This area was selected to ensure a high proportion of women with an ethnic minority background, and because the majority (75-85 %) of pregnant women residing in this area attend the Child Health Clinics for antenatal care. Antenatal care is free of charge in Norway and easily accessible. To facilitate inclusion of ethnic minority women, information material about the study and questionnaires were translated to Arabic, English, Sorani, Somali, Tamil, Turkish, Urdu and Vietnamese and quality checked by bilingual health professionals. The STORK Groruddalen project has been described in detail elsewhere [18]. In short, women were eligible if they 1) lived in the district, 2) planned to give birth at one of the two study hospitals, 3) were <20 weeks pregnant, 4) could communicate in Norwegian or any of the specified languages and 5) were able to provide written consent to participate. Women with pre-pregnancy diabetes or other diseases necessitating intensive hospital follow-up during pregnancy were excluded. In total, 59 % of the included women were of ethnic minority background. The participation rate was 74 % (range: 64-83 % for ethnic groups), and the participating women were found representative for the main ethnic groups of pregnant women attending Child Health Clinics in Oslo [18]. Data from questionnaires, measurements and blood samples were collected at 15 and 28 weeks of gestation, through interviews by specially trained and certified midwives according to the study protocol, assisted by professional interpreters when needed.

### Data collection and variables

#### Primary outcome measure

S-25-hydroxyvitamin D [25(OH)D] was analyzed by competitive radioimmunoassay (DiaSorin, Stillwater, MN, USA) at the Hormone Laboratory, Oslo University Hospital, Aker. The method measures a total value of 25(OH)D (both 25(OH)D_2_ and -D_3_), with intra- and inter-assay coefficients of variation 6 % and 13-16 %, respectively. The laboratory’s normal reference range was 37–131 nmol/l based on the ethnic Norwegian population from the Oslo Health Study [19]. The laboratory is accredited by International Organization for Standardization and is part of the Vitamin D Quality Assessment Scheme, DEQAS. Samples were analyzed consecutively and results were sent to the participant’s midwife.

In this paper, vitamin D deficiency was defined as 25(OH) D <50 nmol/L, and severe vitamin D deficiency was defined as 25(OH) D <25 nmol/L, in accordance with relevant literature [20]. Undetectable concentrations of 25(OH)D (<12 nmol/L) were replaced with “11 nmol/L” in the calculations (*n* = 17).

#### Explanatory factors

Ethnic origin was defined by the participant’s mother’s country of birth[18]. Ethnicity was further categorized into Western Europe (primarily Norway, Sweden and Denmark), South Asia (primarily Pakistan and Sri Lanka), Middle East (primarily Iraq, Turkey, Morocco and Afghanistan), Africa (Somalian being the largest group), East Asia (primarily Vietnam, Philippines and Thailand), and Other (primarily Poland, Russia and Kosovo). Parity was categorized as no children (nulliparous), one child (uniparous) and two or more children (multiparous). Pre-pregnancy body mass index (BMI; calculated from self-reported weight before pregnancy and height measured at inclusion) was categorized as <25 kg/m^2^ and ≥25 kg/m^2^. Education level was categorized as <10 years, 10–12 years and >12 years. Winter season was defined as December to May. According to National Clinical Guidelines for Antenatal Care, the recommendation of vitamin D intake during a normal pregnancy is 10 μg/day [21]. All participants were asked about their intake of vitamin D supplements including prenatal vitamins during the past two weeks at both visits, and self-reported intake was categorized as “ vitamin D ≥10 μg/day” and “no or vitamin D <10 μg/day”. Other variables of interest were age and gestational week at inclusion. Gestational week at inclusion was dichotomized according to the median.

### The recommendation of vitamin D supplementation

Pre-planned, and according to the protocol, women with 25(OH)D less than the laboratory’s lower reference range (<37 nmol/L) at gestational week 15 were provided with written information describing their 25(OH)D D concentration, and they were recommended to consult their General Practitioner (GP) for treatment. Specially, these women were provided a written note advising their GP to prescribe 20 μg (800 IU)/day vitamin D_3_ for 1–3 months if 25(OH)D ranged from 12–37 nmol/L or 30 μg (1200 IU)/day vitamin D_3_ for 3 months if 25(OH)D <12 nmol/L. In addition, 1 g calcium/day was recommended for both groups, and GPs were encouraged to offer follow-up measurements. Due to lack of safety evidence for the fetus and mother for higher doses, and to avoid hypercalcemia in the fetus/newborn, we used a daily dose below the Tolerable Upper Intake Level (for adults 50 μg/d (2000 IU)) when the study was performed [22]. We have no information on the exact dose of vitamin D supplements taken if self-reported intake was >10 μg/day.

### Ethics

The Regional Committee for Medical and Health Research Ethics South East (Ref 2007/894) and the Norwegian Data Inspectorate approved the study protocol. Participation was based on informed, written consent.

### Study sample

Of the 823 women included in the STORK Groruddalen project, 807 (98 %) had 25(OH)D concentrations measured at gestational week 15. At gestational week 28, 20 women had had an abortion or extreme premature delivery, 29 did not re-attend, and 10 women lacked a 25(OH) D value, resulting in a final sample of 748 (91 %) women with two 25(OH)D measurements in this study (Flow chart, Additional file 1: Supplementary Figure).

### Statistics

Descriptive statistics are presented as frequencies, proportions, mean values and standard deviations (SD). All continuous variables were assessed for normality. In Additional file 2: Supplementary table, we report mean values for 25(OH)D for ethnic groups with ≥10 participants. Comparisons of means and proportions were tested by independent and paired t-tests and chi-square tests. Explanatory linear regression models were performed to assess the relationship between ethnicity and the concentration of 25(OH)D, both at inclusion and in gestational week 28, accounting for the following potential confounding factors: gestational week, age, parity, season, education, vitamin D supplements and pre-pregnancy BMI. Due to a non-linear relation with 25(OH)D, pre-pregnancy BMI was dichotomized. Factors with a *p*-value <0.2 in the univariate analysis were included in the multiple regression analyses. Interactions were examined graphically and by adding interaction terms (Western and summer vs non-Western and winter) into the models. Results are presented as regression coefficients (B) with 95 % confidence intervals (CI) and accompanied adjusted R^2^. *P*-values <0.05 were considered statistically significant. SPSS software (Version 22, IBM SPSS statistics, NY, USA) was used for statistical analysis.

## Results

Mean maternal age was 30 (SD: 4.9) years (range 19 to 45) and mean pre-pregnancy BMI 24.6 (SD: 4.8) kg/m^2^. The mean gestational week was 15.4 (SD: 3.6) at inclusion and 28.8 (1.4) at the follow-up visit (Table 1). The ethnic minority women were younger, had higher parity and lower education (25 % reported no, or less than 10 years of schooling) than Western European women. A total of 23 % of ethnic minority women needed an interpreter (21 % reported “poor" or “somewhat poor” skills in the Norwegian language, while 18 % “medium” skills). Reported reasons for migrating to Norway were work or studies (9.1 %), refugees or asylum seekers (18 %) and family immigration (73 %). About two thirds of Western European and East Asian women reported using vitamin D supplements regularly at inclusion compared to 50 % of other ethnic groups.

**Table 1:** Characteristics of the total cohort of pregnant women at inclusion and at gestational week 28 stratified by geographic region. Numbers are frequencies (%) and means (SD)

	Total		Western Europe^a^		South Asia		Middle East		Sub-Saharan Africa		East Asia		Other	
	n = 748		n = 304		n = 189		n = 113		n = 51		n = 40		n = 51	
	100 %		41 %		25 %		15 %		6.8 %		5.3 %		6.8 %	
Pre-pregnancy maternal status														
Years of maternal age; mean (SD)	29.9 (4.9)		30.9 (4.5)		28.7 (4.5)		29.6 (5.5)		28.1 (5.2)		31.1 (4.5)		29.3 (5.1)	
Parity; n (%)														
Para 0	340 (46)		156 (51)		78 (41)		39 (35)		22 (43)		17 (42)		28 (55)	
Para 1	259 (34)		113 (37)		63 (33)		39 (35)		11 (22)		16 (40)		17 (33)	
Para ≥2	149 (20)		35 (12)		48 (25)		35 (30)		18 (35)		7 (18)		6 (12)	
ᅟEducation level; n (%)^b^														
ᅟᅟ<10 years	119 (16)		10 (3.3)		33 (18)		41 (36)		22 (43)		8 (20)		5 (9.8)	
ᅟᅟ10-12 years	296 (40)		93 (30)		95 (50)		51 (45)		22 (43)		16 (40)		19 (37)	
ᅟᅟ>12 years	327 (44)		199 (66)		60 (32)		19 (17)		7 (14)		16 (40)		26 (51)	
ᅟPre-pregnancy BMI^b^ (kg/m^2^); mean (SD)	24.6 (4.8)		24.6 (4.8)		23.7 (4.1)		25.8 (5.0)		26.1 (6.1)		22.2 (3.4)		24.9 (5.4)	
Status at inclusion														
ᅟGestational week; mean (SD)	15.4 (3.6)		14.5 (2.4)		15.9 (4.1)		15.6 (3.3)		17.9 (5.4)		16.4 (4.3)		15.5 (3.4)	
ᅟSeason of blood sample; n (%)														
ᅟᅟWinter	395 (53)		158 (52)		92 (49)		70 (62)		32 (63)		22 (55)		21 (41)	
ᅟVitamin D supplements; n (%)^b^														
ᅟᅟNo or <10 μg daily past two weeks	307 (41)		104 (34)		93 (49)		55 (49)		25 (49)		12 (30)		18 (35)	
Status at gestational week 28														
ᅟGestational week; mean (SD)	28.8 (1.4)		28.7 (1.3)		28.7 (1.4)		29.0 (1.6)		29.0 (1.5)		28.7 (1.2)		29.0 (1.7)	
ᅟWeight gain (week 28 minus 15); mean (SD)	6.6 (3.2)		6.7 (2.7)		6.5 (3.3)		6.7 (3.6)		4.6 (3.0)		6.2 (2.5)		8.0 (3.9)	
ᅟSeason of blood sample; n (%)														
ᅟᅟWinter	410 (55)		178 (59)		97 (51)		65 (57)		28 (55)		17 (57)		25 (49)	
ᅟVitamin D supplements; n (%)^b^														
ᅟᅟNo or <10 μg daily past two weeks	243 (33)		115 (38)		41 (22)		37 (33)		20 (40)		14 (35)		16 (31)	

Notes:

Countries with ≥10 individuals are listed: 304 women from Western Europe, primarily from Norway (*n* = 278), 189 women from South Asia, primarily from Pakistan *n* = 120), Sri Lanka (*n* = 56) and India (*n* = 12), 113 women from the Middel East, primarily from Iraq (n = 34), Morocco (*n* = 27), Turkey (n = 25), Afghanistan (*n* = 12), 51 women from Sub-Saharan Africa, primarily from Somalia (*n* = 32), 40 women from East Asia, primarily Vietnam (*n* = 17), Philippines (*n* = 12).

^a^Including 3 women from USA and Canada

^b^Incomplete data on the variables because of missing values for 6–12 women

At gestational week 28, the proportion of South Asian and Middle Eastern women who had used vitamin D supplements ≥10 μg/day over the past two weeks was higher than at gestational week 15 (*p* < 0.01 and *p* < 0.05, respectively). No change in the proportion using vitamin D supplements was observed among the other ethnic groups (Table 1). No significant differences between the study sample and the 75 excluded women were found for ethnicity, age, gestational week (both visits), parity, pre-pregnant BMI and education (data not shown).

### Vitamin D status in early pregnancy at gestational week 15

**Fig. 1:**
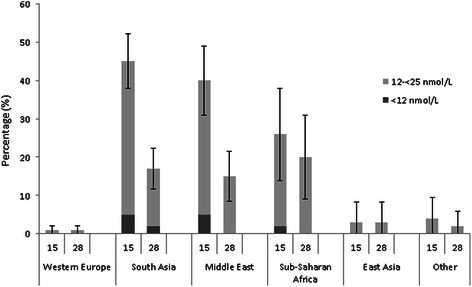
Proportions of participants with levels of 25(OH)D 12- < 25 nmol/L and <12 nmol/L at gestational week 15 and 28

The 25(OH)D concentration at inclusion ranged from <12 to 148 nmol/L, with large differences in mean values between the ethnic groups (Table 2). Mean values for countries with ≥10 participants are provided in Additional file 2: Supplementary table. The prevalence of 25(OH)D <50 nmol/L differed between the ethnic groups as follows: South Asia: 84 %, the Middle East: 79 %, Sub-Saharan Africa: 75 %, East Asia: 43 % and Western Europe: 20 %. For severe deficiency (25(OH)D <25 nmol/L), corresponding numbers were: South Asia: 45 %, the Middle East: 40 %, Sub-Saharan Africa: 26 %, East Asia: 2.5 % and Western Europe: 1.3 %, representing 20 % of the total cohort (Fig. 1).

**Table 2:** Descriptive vitamin D at inclusion (gestational week 15) and at gestational week 28. Crude mean (SD) Vitamin D [25(OH)D in nmol/l] levels according to potential explanatory factors

	At inclusion		At gestational week 28	
	*n* = 748		*n* = 748	
	Mean	(SD)	Mean	(SD)
Overall mean 25(OH)D	50	(27)	59	(29)
Age at inclusion				
≤30 years	47	(25)	55	(27)
>30 years	54	(28)	62	(30)
Parity (Para 0 ref)	53	(26)	61	(29)
Para 1	52	(28)	59	(27)
Para ≥2	42	(25)	52	(30)
Western Europe (ref)	69	(24)	72	(28)
South Asia	32	(19)	46	(23)
Middle East	34	(20)	51	(29)
Sub-Saharan Africa	38	(18)	45	(25)
East Asia	51	(17)	53	(19)
Other	56	(21)	63	(26)
Season of blood sample				
Summer	56	(29)	60	(29)
Winter	46	(24)	57	(27)
Education level (>12 years ref)	60	(27)	67	(27)
10-12 years	46	(25)	56	(29)
<10 years	37	(20)	44	(23)
Vitamin D supplements				
≥10 μg^a^	57	(26)	61	(28)
No or <10 μg	42	(26)	53	(29)
Pre-pregnancy BMI; kg/m^2^ (normal weight (<25) ref)	51	(27)	59	(29)
Overweight (≥25/<30)	51	(27)	61	(31)
Obesity (≥30)	48	(24)	54	(24)

^a^Intake of ≥10 μg vitamin D daily past two weeks

In early pregnancy, the values of 25(OH)D differed between the ethnic groups and remained significant after adjustment for age, parity, season, education and intake of vitamin D supplements (Table 3). Women from South Asia, the Middle East and Sub-Saharan Africa had mean values that were −28 (95 % CI: −33, −23), −24 (−29, −18) and −20 (−27, −13) nmol/L lower than in Western European women, respectively. In addition, education, intake of vitamin D supplements and season were independently associated with 25(OH)D concentrations at inclusion. The effect of season on 25(OH)D differed by ethnicity (interaction term, *p* < 0.01). Western European women had a higher concentration of 25(OH)D during summer compared to winter, while no seasonal difference was observed among ethnic minority women, and their mean values were lower than Western Europeans.

**Table 3:** Linear regression analysis with vitamin D [25(OH)D] at inclusion and gestational week 28 as dependant variables

	At inclusion									At gestational week 28								
	Univariate analysis					Multiple analysis				Univariate analysis					Multiple analysis			
						n = 735, R^2^ adj. = 0.46									n = 730, R^2^ adj. = 0.24			
Independent variable	R^2^ adj.	B	95 % CI			B	95 % CI			R^2^ adj.	B	95 % CI			B	95 % CI		
			Lower	Upper			Lower	Upper				Lower	Upper			Lower	Upper	
Gestational week at inclusion (<15 ref)	−0.001									0.01								
ᅟ≥15		−0.56	−4.4	3.3							**−6.4**	**−10**	**−2.3**	**	**−4.6**	**−8.3**	**−0.92**	*
Age at inclusion	0.03	**0.94**	**0.55**	**1.3**	**	0.14	−0.21	0.49		0.03	**1.1**	**0.64**	**1.5**	**	**0.70**	**0.26**	**1.1**	**
Parity (para 0 ref)	0.02									0.01								
ᅟPara1		−1.2	−5.4	3.1		0.24	−3.1	3.6			−2.8	−7.4	1.8		**−4.4**	**−8.7**	**−0.22**	*
ᅟPara ≥2		**−11**	**−17**	**−6.3**	**	−3.2	−7.6	1.3			**−9.3**	**−15**	**−3.8**	**	−5.4	−11	0.25	
Geographic region (Western Europe ref)	0.38									0.16								
ᅟSouth Asia		**−37**	**−40**	**−33**	**	**−28**	**−33**	**−23**	**		**−26**	**−31**	**−21**	**	**−17**	**−23**	**−11**	**
ᅟMiddle East		**−34**	**−39**	**−30**	**	**−24**	**−29**	**−18**	**		**−21**	**−26**	**−15**	**	**−9.5**	**−16**	**−2.9**	**
ᅟSub-Saharan Africa		**−31**	**−37**	**−25**	**	**−20**	**−27**	**−13**	**		**−26**	**−34**	**−19**	**	**−12**	**−20**	**−3.1**	**
ᅟEast Asia		**−17**	**−24**	**−10**	**	**−11**	**−18**	**−3.9**	**		**−19**	**−28**	**−11**	**	**−11**	**−20**	**−1.6**	*
ᅟOther		**−13**	**−19**	**−6.4**	**	**−7.6**	**−14**	**−0.90**	*		**−9.0**	**−17**	**−1.3**	*	−1.4	−9.6	6.9	
Season (Summer ref)	0.03									0.01								
ᅟWinter		**−9.9**	**−14**	**−6.1**	**	**−5.2**	**−8.9**	**−1.4**	**		**−5.7**	**−9.8**	**−1.6**	**	−1.3	−6.1	3.4	
Geographic region*Season^a^						**−11**	**−16**	**−4.8**	**						**−15**	**−22**	**−7.4**	**
Education level (>12 y ref)	0.11									0.08								
ᅟ10-12 year		**−14**	**−18**	**−10**	**	**−3.7**	**−7.1**	**−0.30**	*		**−11**	**−15**	**−6.8**	**	−1.8	−6.1	2.5	
ᅟ<10 year		**−23**	**−28**	**−17**	**	**−5.2**	**−10**	**−0.31**	*		**−23**	**−29**	**−17**	**	**−8.6**	**−15**	**−2.5**	**
Vitamin D supplements (≥10μg^b^ ref)	0.08									0.02								
ᅟNo or <10 μg		**−15**	**−19**	**−11**	**	**−11**	**−14**	**−7.7**	**		**−8.2**	**−13**	**−3.8**	**	**−9.8**	**−14**	**−5.8**	**
Pre-pregnancy BMI (<25 kg/m^2^ref)	−0.001									−0.001								
ᅟ≥25 kg/m^2^		−0.76	−4.7	3.2							−0.11	−4.4	4.2					

^a^The effect of season on 25(OH)D differed by ethnicity: the interaction term is "1" for records with both non-Western and winter and "0" for Western and summer (ref)

^b^Intake of ≥10 μg vitamin D daily past two weeks

Bold numbers indicate *p* < 0.05 (*p < 0.05, ** *p* < 0.01)

### Vitamin D status at gestational week 28

At the beginning of the third trimester, the prevalence of 25(OH)D <50 nmol/L among the largest ethnic minority groups was lower than at study inclusion: South Asia: 62 %, the Middle East: 58 % and Sub-Saharan Africa: 63 %. The prevalence of 25(OH)D <25 nmol/L was also lower: South Asia: 17 %, Middle East: 15 %, Sub-Saharan Africa: 20 % (Fig. 1).

Mean 25(OH)D values were −17 (95 % CI: −23, −11), −10 (−16, −2.9) and −12 (−20, −3.1) nmol/L lower in South Asians, Middle Easterners and Sub-Saharan Africans compared with Western Europeans after adjustments for gestational week at inclusion, age, parity, season, education and vitamin D supplements (Table 3). In addition, age, parity, education, intake of vitamin D supplementation and gestational week at inclusion were independently associated with 25(OH)D concentrations. The effect of season on 25(OH)D differed by ethnicity (interaction term, *p* < 0.01) at gestational week 28.

A sensitivity analysis to evaluate the effect of the interaction term was performed. When excluding the geographic region*season interaction term from the multiple analyses (Table 3), the coefficients for ethnic minority groups and season were reduced by the same amount as the interaction-coefficient, while the coefficients for the remaining factors were about the same.

### Vitamin D status in women who were recommended supplementation in early pregnancy

**Fig. 2:**
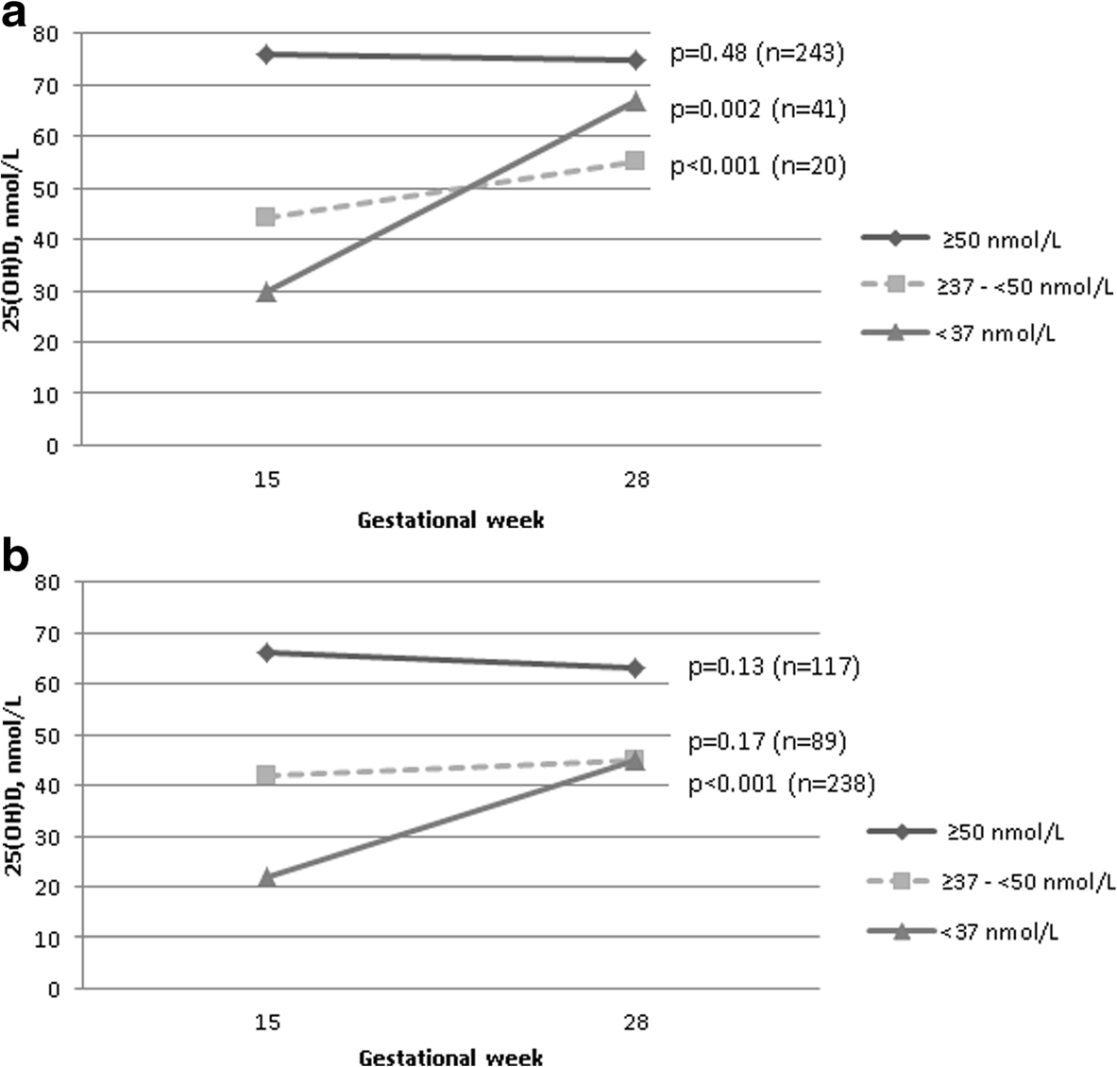
**a**. Change in unadjusted mean 25(OH)D of Western European women at gestational week 15 and 28 - stratified for baseline levels (paired t-test). Women with <37 nmol/L at baseline were recommended 20 μg or 30 μg vitamin D daily. **b**. Change in unadjusted mean 25(OH)D of ethnic minority women at gestational week 15 and 28 - stratified for baseline levels (paired t-test). Women with <37 nmol/L at baseline were recommended 20 μg or 30 μg vitamin D daily

In total, 258 women (35 %) had values <37 nmol/L at inclusion and were recommended to consult their GPs for supplements. Only 37 % of these women reported an intake of ≥10 μg/day of vitamin D supplements, but at gestational week 28, this proportion had increased to 73 % (*p* < 0.01). Among the 67 women reporting no or <10 μg/day at this time point, 11 women had 25(OH)D >50 nmol/L and 26 women (10 % of the group recommended supplementation and 3.5 % of the total cohort), had 25(OH)D <25 nmol/L. In parallel to the increased use of supplements in this group, the mean 25(OH)D increased from 23 (SD:7.8) to 47 (27) nmol/L (*p* < 0.01). No changes in mean 25(OH)D were observed among women with baseline values >50 nmol/L, while a slight increase in mean 25(OH)D was observed in women with baseline values ranging from 37–50 nmol/L. Corresponding results are presented for Western European women (Fig. 2a) and the merged ethnic minority groups (Fig. 2b). The mean change in 25(OH)D from GW 15 to 28 was 24 nmol/L in the group recommended supplementation compared to 0.1 nmol/L in the group with levels ≥37 nmol/L (*p* < 0.01).

## Discussion

### Main findings

In this cohort study we found a high prevalence of vitamin D deficiency (<50 nmol/L) in early pregnancy among women from South Asia, Middle East and Sub-Saharan Africa, and severe deficiency (<25 nmol/L) was prevalent among women from these regions. Independent risk factors for low 25(OH)D values in early pregnancy included ethnic origin from South Asia, the Middle East and Sub-Saharan Africa, low intake of supplements, low education, and examination during winter. Among women who were recommended to consult their GPs for supplementation, use of supplements and levels of 25(OH)D increased substantially more than in women with values ≥37 nmol/L who not were exposed to this recommendation.

### Strengths and limitations

The major strength of the study is that we followed a large number of healthy women representative for the main ethnic groups of pregnant women in Oslo through pregnancy. The questionnaires were translated to eight languages and data collection methods were adapted to facilitate inclusion of ethnic minorities and even illiterate women, who are often excluded in research [18]. Professional interpreters were used to ensure the quality of the interview administered questionnaire data. We were able to include a relatively wide range of explanatory factors, and we collected data at two time points in pregnancy. The blood samples were analyzed at the same laboratory with standardized methods.

However, there are also weaknesses. This was not a randomized, controlled trial, but was evaluated by a pre-post test design. We do not directly know the impact of the letter from the study personnel to the GPs, nor do we know the participants’ level of adherence to the recommendations from the GPs. However, 73 % reported use of ≥10 μg/day of vitamin D supplements at the post-test, and only 3.5 % still had severe deficiency, compared to 20 % at inclusion. We lack information on other variables affecting the concentration of 25(OH)D, including the degree of concealing clothes, skin pigmentation and sunlight exposure.

### Interpretation

Our findings are in line with others confirming widespread vitamin D deficiency during pregnancy among ethnic minorities [2, 3, 11, 23, 24], although some studies from Northern Europe have found a comparatively higher prevalence and more severe deficiency than observed in our study [6, 25]. Recent studies from the same part of Oslo may have increased awareness of vitamin D deficiency among health personnel [26–28], which may have contributed to the somewhat lower prevalence of vitamin D deficiency in ethnic minorities in our study. However, direct comparison of 25(OH)D values between studies are hampered by different methodologies such as inconsistent definitions of vitamin D deficiency, laboratory measurements and degree of adjustment for possible confounders.

Most studies that we identified were cross-sectional, and few adjusted for intake of vitamin D supplements, education, parity and seasonal variance [5, 29]. At high latitude, seasonal variance is an important explanatory factor, as the sun is not capable of producing vitamin D by skin radiation during winter time [30]. Some ethnic minority groups have other traditions of sun exposure than Western European women. However, we did not identify other studies from Europe testing for the interaction of ethnicity and season, while no interaction was found in a study from the US [31]. We found that low education was an independent risk factor for low values of 25(OH)D, but did not identify other studies from Europe which investigated education as an explanatory factor, although one study from the Netherlands found that less educated women had lower 25(OH)D levels compared to high educated women[32]. Our data also indicated that low self-reported intake of vitamin D supplements was an independent risk factor for vitamin D deficiency, also found in an Irish study [3]. In contrast to several prior studies, BMI was not a significant risk factor in our sample [5, 11, 33].

The debate about the optimal dose of vitamin D supplementation during pregnancy is ongoing. Guidelines from Scandinavia and UK recommend all pregnant women supplement intake with 10 μg (400 IU) vitamin D daily to prevent vitamin D deficiency [21, 34, 35]. The guidelines from the Institute of Medicine (IOM)[36], the Endocrine Society Clinical Practice [37], the WHO [38] and the Canadian Pediatric Society [39] differ. There is no consensus of the optimal concentration of 25(OH)D in pregnancy [40, 41]. There is no doubt that low infant 25(OH)D increases the risk of skeletal diseases such as rickets and hypocalcemia, but the effect of maternal deficiency on the fetus is not clear. Concerns about the impact of low 25(OH)D concentrations during pregnancy on fetal development is present, as low levels have been associated with preterm birth, low birth weight, bone mass and postnatal calcium concentrations, as well as preeclampsia and gestational diabetes in the mother [11, 42].

Several studies among pregnant women have showed a marked increase in 25(OH)D with daily supplements of vitamin D [11, 24, 43, 44]. Although there may be changes to the pharmacokinetics of 25(OH)D during pregnancy, such as increased binding proteins and fluid redistribution, most studies report that 25(OH)D concentration is relatively constant, at least during the first two trimesters [4, 45]. However, some evidence suggests vitamin D binding protein may increase and the free fraction of 25(OH)D may decrease [46, 47].

In women who were recommended daily vitamin D supplements of 20 μg or 30 μg due to low values in early pregnancy, we observed an increase in self-reported use of supplements at the follow-up visit. We similarly observed a substantial increase in 25(OH)D concentrations and a reduced prevalence of 25(OH)D <25 nmol/L. The small increase of 25(OH)D observed among women with a slight deficiency at baseline who did not receive the recommendation, may be related to usual practices in Norway of routinely recommending a daily intake of 10 μg vitamin D as part of antenatal care [21]. No similar increase was observed among ethnic minority women with a slight deficiency, probably indicating inadequate delivery of the message or less compliance in these groups. Compared with no change or a small increase in 25(OH)D concentrations in women with adequate levels or mild vitamin D deficiency, this differential change may indicate a beneficial effect of the recommendations. However, it should be noted that severe vitamin D deficiency was still found in 15-20 % of the high risk groups. Although the results must be interpreted with caution because we did not conduct a randomized controlled trial, the observed increase in 25(OH)D levels in our study is unlikely to be attributable to physiological changes during pregnancy or regression to the mean only. Supplementation with a higher dose of vitamin D than 20 μg or 30 μg would likely be sufficient to achieve acceptable 25(OH)D concentration in pregnancy.

## Conclusion

We found that vitamin D deficiency was prevalent among South Asian, Middle Eastern and African women in early pregnancy. Ethnic minority background, low intake of supplements, low education and winter season were independent risk factors for vitamin D deficiency. After identifying participants with low 25(OH)D values at study inclusion, and recommending treatment with daily moderate doses of vitamin D, the daily intake of supplements significantly increased and the prevalence of severe vitamin D deficiency was substantially reduced. This cost-effective, and easy-to-administer recommendation likely contributed the most to the improved vitamin D status later in pregnancy for these women.

## Additional files

Additional file 1:
**Supplementary figure.** Flow-chart. (TIF 64 kb) Additional file 2:
**Supplementary table.** (XLSX 11 kb)

## Abbreviations

25(OH)D: Serum-25-hydroxyvitamin D
BMI: body mass index
CHC: Child Health Clinics
DEQAS: Vitamin D Quality Assessment Scheme
GP: General Practitioner
GW: Gestational week
IOM: Institute of Medicine
IU: International Units
R^2^: Adjusted R-square
SD: Standard deviation
SPSS: Statistical package for the social science
UK: United Kingdom
US: United States of America
WHO: World Health Organization
