# Supplementary figures and images for: Vitamin D deficiency and supplementation in pregnancy in a multiethnic population-based cohort
Source: BMC Pregnancy Childbirth. 2016 Jan 19;16:7. doi: 10.1186/s12884-016-0796-0 (PMC4719746; doi:10.1186/s12884-016-0796-0)

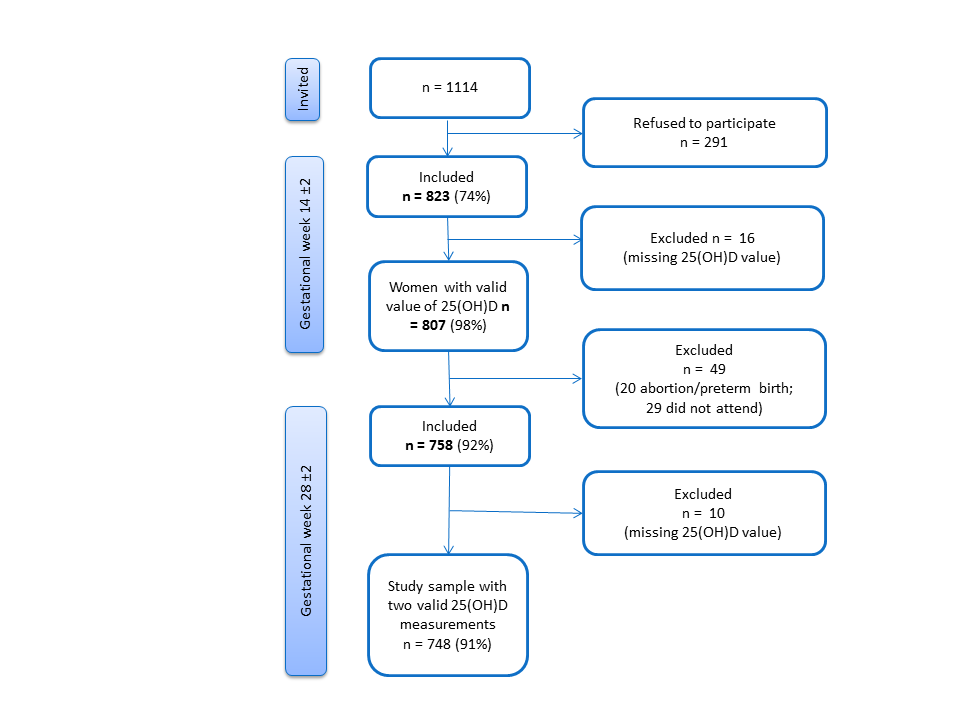

Supplement: Additional file 1: — Supplementary figure. Flow-chart. (TIF 64 kb) [file 12884_2016_796_MOESM1_ESM.tif]
